# Physiological Incest

**Published:** 1860-04

**Authors:** W. Boyd Powell

**Affiliations:** Covington, Ky.


### ARTICLE 5.

For the Chicago Medical Journal.

### PHYSIOLOGICAL INCEST.

BY W. BOYD POWELL M. D., COVINGTON, KY.

Messrs. Editors:—By the above title I designate marriage in contravention of physiological law.

There obtains in society a very general conviction that consanguine marriages deteriorate the species by entailing on progeny idiotcy, other mental imperfections and constitutions so depraved as readily to develop scrofulous forms of disease; and yet it is strongly to be suspected that the truth of this general conviction has never been established. Some fifteen years ago I was induced to suspect the existence of a physiological incompatibility between the sexes in relation to progeny and thus I was induced to adopt a course of action and careful observation of progenitors and progeny, and the result has been a thorough conviction that my suspicion was well founded,—that a physiological incompatibility in relation to progeny obtains very frequently between the sexes, and it consists in this:—certain of the human temperaments are incompatible in relation to progeny. This *a priori* appears to be much more probable than that consanguinity should produce an incompatibilty, because temperament is a positive condition and therefore may become an agent; but consanguinity is merely a relation which can exert no agency.

I nominate this physiological incompatibility, physiological incest; and to the extent of my knowledge it is the only incest nature has established; and to this incest do I attribute all the idiots, much of the insanity, all genuine phthisis, and all scrofulous forms of disease, half of the juvenile mortality, and a large percentage of the premature adult mortality which now afflict the human race. I admit however, that these evils do attend occasionally consanguine progenitors, and as consanguine parties are not physiologically exceptions to the race, it follows that they are as vulnerable to physiological incest as other parties, but I deny that consanguinity has any agency in this relation.

That system of the temperaments which I adopt, with some modification, is that which has descended to us, probably from Hippocrates or Galen. It embraces four temperaments, namely: the Sanguine, the Bilious, the Lymphatic, and the Melancholic. The description which has been had of the Melancholic so strongly indicates a pathological condition that physiologists seem to have, by a common consent, ceased to regard it as a physiological one; nevertheless, early in my investigations of the temperaments, I became convinced that humanity was distinguished by four constitutional conditions or temperaments, and after seven years of unceasing observation, I discovered an individual whom I supposed to represent the fourth temperament, and I nominated it the Encephalic, and no fact has since come to my knowledge to induce me to suspect that I had adopted an erroneous conclusion. But I strongly suspect that the ancients observed the same condition and nominated it the Melancholic, but if they did they left no evidence that they comprehended either its anatomical or physiological condition. The Encephalic temperament is distinguished by a large cerebrum and a small cerebellum, consequently all the functions incidental to life, both animal and vegetative, are feebly manifested.

The four elementary temperaments, by their combinations, produce ten more, making, in all, fourteen, and for the purpose of convenient reference, I will tabulate them.

THE ELEMENTARY TEMPERAMENTS.

1. Sanguine temperament.
2. Bilious "
3. Lymphatic "
4. Encephalic "

THE BINARY COMBINATIONS OF THE TEMPERAMENTS.

5. Sanguine bilious temperament.
6. Sanguine lymphatic "
7. Sanguine encephalic "
8. Bilious lymphatic "
9. Bilious encephalic "

TERNARY COMBINATIONS OF THE TEMPERAMENTS.

10. Sanguine bilious lymphatic temperament.
11. Sanguine bilious encephalic "
12. Sanguine encephalic lymphatic "
13. Bilious encephalic lymphatic "

QUARTERNARY COMBINATION OF THE TEMPERAMENTS.

14. Sanguine encephalic bilious lymphatic temperament.

*Application of the Temperaments to the Elucidation of Physical Incest.*—All the temperaments are incompatible with themselves, but 1, 2, and 5 are less so than the others; and an alliance between either two of 1, 2, and 5, is incompatible; but 1, 2, and 5, are compatible with all the others.

But no other than a highly incestuous marriage can be made between any two of 3, 4, 6, 7, 8, 9, 10, 11, 12, 13, and 14.

If an alliance between 3 and 4 produced any progeny, which I think to be very improbable, it would be dead born. But such a marriage I have never seen.

Death in infancy attends the progeny of an alliance between persons indicated by 6 and 7. And an alliance between persons indicated by 7 and 8, is equally unfortunate.

The progeny of an alliance between 7 and one of same, will die in infancy of hydrocephalous, or tubercular meningitis.

The progeny of progenitors indicated by 8 and 9 will die young of scrofulous forms of disease.

The progeny of progenitors indicated by 9, will be idiotic.

The progeny of progenitors indicated by 9 and 14, will, some time in early life, become insane.

The progeny of progenitors indicated by 8 and 11, will die of tubercular disease of the lungs or mesenteric glands.

Two-thirds of the progenitors indicated by 13 will be dead born, and the other third will die during infancy.

Progenitors indicated by 12 and 13 will be attended with similar results.

P. S.—I have given so much attention to this subject, that I can at sight distinguish physiologically incestuous parties, and can teach any other person of respectable capacity to do the same.

Now, Messrs. Editors, do you entertain a doubt as to my having made the discovery herein claimed? If you do, review your own practice for a few minutes, and you will be able to call up cases that will sustain me. Have you not treated scrofulous forms of disease in the families of sound and healthy progenitors, those, too, whose constitutions contra-indicated any scrofulous taint? Such is the depopulating influence of this incest, that were emigration to our country to cease, I believe it would, in two hundred years, depopulate our country. If this contribution find a place in your journal please favor me with a copy.

I will conclude by giving you a patent fact. Dr. Patterson, Superintendent of the Idiotic Asylum at Columbus, Ohio, in response to my inquiries, says that he has carefully inquired as to the progenitors of the idiots brought to the institution, and has not been able to learn that more than two per centum of them were of consanguine progenitors.

